# Measurement of the burdens of neonatal disorders in 204 countries, 1990–2019: a global burden of disease-based study

**DOI:** 10.3389/fpubh.2023.1282451

**Published:** 2024-01-09

**Authors:** Juan Xin, Yiwen Luo, Wanwan Xiang, Sijing Zhu, Hui Niu, Jiayuan Feng, Landi Sun, Boxing Zhang, Xihui Zhou, Wenfang Yang

**Affiliations:** ^1^Department of Obstetrics and Gynecology, Maternal and Child Health Center, The First Affiliated Hospital of Xi’an Jiaotong University, Xi’an, China; ^2^Continuing Education and Training Center, The First Affiliated Hospital of Xi'an Jiaotong University, Xi’an, China; ^3^School of Public Health, Jiaotong University Health Science Center, Xi’an, China; ^4^Department of Pediatrics, The First Affiliated Hospital of Xi’an Jiaotong University, Xi’an, China

**Keywords:** neonatal disease burden, incidence, death rate, disability-adjusted life years, global trend

## Abstract

**Background:**

Neonatal disorders are facing serious public health challenges. Previous studies were based on limited data sources and had a narrow geographical scope. We aim to understand the trends of alteration in the burden of neonatal disorders from 1990 to 2019 in 204 countries and territories.

**Methods:**

Data were investigated from the Global Burden of Disease Study 2019. First, we visualized the burden of neonatal disorders using the number of cases and the age-standardized incidence rate (ASIR), death rate (ASDR), and disability-adjusted life years (ASR-DALYs) from 1990 to 2019. Second, estimated annual percentage changes (EAPCs) were used to evaluate the temporal trends of disease burden during different periods. Finally, the sociodemographic index (SDI) and human development index (HDI) were used to determine whether there exists a correlation between socioeconomic development level, human development level, and potential burden consequences.

**Results:**

Overall, in the past 30 years, the ASIR trends have remained relatively steady, whereas the ASDR and ASR-DALYs have declined. However, the burden of neonatal disorders varied greatly in various regions and countries. Among 21 regions, the ASIR trend had the largest increase in Central Latin America (EAPC = 0.42, 95%CI = 0.33–0.50). Conversely, the ASDR and ASR-DALYs experienced the largest decrease in Central Europe (EAPC = −5.10, 95%CI = −5.28 to 4.93) and East Asia (EAPC = −4.07, 95%CI = −4.41 to 3.73), respectively. Among 204 countries, the ASIR (EAPC = 3.35, 95%CI = 3.13–3.56) trend in Greece displayed the most significant increase, while the ASDR (EAPC = 1.26, 95%CI = 1.01–1.50) and ASR-DALYs (EAPC = 1.26, 95%CI = 1.03–1.49) trends in Dominica experienced the most substantial increase. Furthermore, there was a strong correlation between the EAPCs in ASIR, ASDR, ASR-DALYs, and SDI or HDI in 2019, with some exceptions. In addition, countries with elevated levels of HDI experienced a faster increase in ASDR and ASR-DALYs for neonatal disorders.

**Conclusion:**

Although the burden of neonatal disorders shows a downward trend from 1990 to 2019, it is still not optimistic. It is necessary to implement a multi-pronged approach to reduce the increasing burden of neonatal disorders.

## 1 Introduction

Neonatal disorders are an important global health problem. According to the report by the United Nations Children’s Fund (UNICEF), approximately 2.7 million neonates die within 28 days of birth globally every year, which indicates that there is a generalized need to raise awareness of neonatal disorders along the entire global spectrum. In the last 30 years, although there has been accelerated progress in reproductive, maternal, newborn, and child health (RMNCH) and the trend in death rates for children aged 5 years and below has significantly decreased, this increased survival explained that deaths in children under 5 mainly occurred in the 4 weeks before life, i.e., the neonatal period (1). To date, neonates are still an unheeded age group in the pursuit of national health coverage. Moreover, as the birth population slightly increases and the population structure, disease spectrum, and social and economic development transform, neonatal disorders have shown differentiation and complexity, it is necessary to have a thorough, systematic, and timely comprehension of the burden caused by neonatal diseases.

At present, the study on the burden of neonatal disorders is mostly focused on a single health problem or independent risk factors. Although past research has reported the global incidence and death rates of neonatal disorders (2), the continuous changes and disability-adjusted life years (DALYs) of neonatal disorders need to be clarified. Their geographical distribution and long-term tendencies have not been explained, and the association between socioeconomic status and human development index in the regional and national contexts also needs to be further clarified.

It is well known that neonatal disorders directly increase the mortality risk of neonates and children under 5 (3), indirectly increase maternal death rates, and can also affect the cognitive, motor, behavioral, and socio-emotional development of children in the later stages, especially the development of the nervous system (4). Currently, approximately 7,000 neonates also die every day, accounting for 47% of all deaths in children under 5 (5). Our team’s previous research has also discovered that exposure during pregnancy to air pollution (especially PM_2.5_ and NO_2_) links to adverse pregnancy outcomes, neonatal neurodevelopment, and low hemoglobin during the third trimester (6–8), which may also lead to the occurrence of neonatal diseases. It can be seen that the cause of neonatal disorders is the result of the interaction of multiple factors. Therefore, understanding the global trend and the temporal change of neonatal disorders is very important for tracking the progress of neonatal survival objectives, strengthening child healthcare and health promotion, and developing national medical policy.

The Global Burden of Disease (GBD) research is a good opportunity to effectively grasp the policy intervention focus of neonatal health under the new situation. In this research, the age-standardized rates (ASRs) of incidence (ASIR), death (ASDR), and DALYs (ASR-DALYs) for neonatal disorders were evaluated at global, regional, and country levels and according to the level of economic development from 1990 to 2019 so as to clarify the health status and major threats of neonatal disorders under the new situation, make global and regional targeted intervention a priority for neonatal disorder management, and reduce the burden of neonatal disorders.

## 2 Methods

### 2.1 Overview

This study strictly followed the Guidelines for Accurate and Transparent Health Estimates Reporting (GATHER) standards (9) and showed the progress, experience, and challenges of different countries in controlling and preventing neonatal disorders. Neonatal disorders in this study refers to the following five categories: premature birth, brain damage resulting from suffocation and injury during birth, neonatal septicemia and infections, hemolytic disorders and neonatal jaundice, and other illnesses that affect newborns. The GBD 2019 database diagnoses of neonatal disorders were based on the International Classification of Diseases, 10th Edition (ICD-10) and 9th Edition (ICD-9). This research did not involve humans and/or animals and did not require ethical approval or informed consent.

### 2.2 Data sources

The GBD study conducted a global comprehensive assessment of health losses for 329 diseases across 204 countries and 21 regions. These diseases were categorized into 21 regions based on epidemiological similarity and geographic proximity. Our study was based on the 2019 GBD data, which were extracted according to the GBD operation guidelines, and options for disease type, sex, and disease burden were set. Neonates were defined as infants within 28 days after birth. We used the online data query tool of the Global Health Data Exchange (GHDx), available at http://ghdx.healthdata.org/gbd-results-tool, to examine the data sources related to the research topic. Data on the frequency of incidence, death rates, and DALYs of neonatal disorders were acquired by us. Furthermore, we calculated ASRs for the years between 1990 and 2019 in light of gender, region, and country. Five different sociodemographic index (SDI)-based groups were included: low, low-middle, middle, high-middle, and high, based on the SDI in 2019. The SDI was a new development classification indicator proposed by the Institute for Health Metrics and Evaluation (IHME) at the University of Washington, along with the results of the 2015 Global Burden of Disease Study. The SDI was an amalgamated indicator that considered a nation’s income *per capita* distributed over time, mean years of schooling, and the rate of fertility in women below the age of 25 (9, 10). It can better reflect the disease burden and the degree of healthy development. The scale of the metric ranged from 0 to 1, with 0 indicating the poorest combination of the three indicators and 1 indicating the most affluent (11). Meanwhile, we also gathered the values for the Human Development Index (HDI). The HDI was an indicator proposed by the United Nations Development Program (UNDP) in the 1990 Human Development Report to measure the level of economic and social development of United Nations member states. The HDI for 2019 could represent the medical level of each country.

### 2.3 Statistical analysis

ASIR, ASDR, and ASR-DALYs and their corresponding estimated annual percentage changes (EAPCs) were calculated to demonstrate the burden of neonatal disorders. ASRs refer to the ratio per 100,000 population following the standardization of the global age structure. The purpose of ASRs was to eliminate the impact of age distribution on the incidence, death, or DALYs within a population. Incidence refers to the number of new cases per 100,000 people in a specific period. The death rate was calculated as the number of fatalities per 100,000 people within a designated time frame. DALYs was a measure that combined the years lived with disability (YLDs) and years of life lost (YLLs) per 100,000 population. YLDs were approximated by multiplying the number of individuals affected by a specific disease or injury by the disability weight assigned to that condition (11). YLLs involved subtracting the age at death from the maximum expected lifespan at that age (11). ASRs (age-standardized incidence/death/DALYs) were reckoned by the following Equation 1 (12):


(1) $$ASR=\frac{\sum_{i=1}^{A}a_{i}w_{i}}{\sum_{i=1}^{A}w_{i}}\times 100,000$$

In the above formula, the numerator was the sum of products obtained by multiplying the age group-specific rates by the count of individuals within the same age group of the selected standard population group, and the denominator was calculated by adding standardized population weights. More importantly, the ASR trends could well reflect the changes in disease models and disease hazards among the crowd. Thus, this study could assess the rationality of current measures to prevent and control neonatal disorders through the analysis of the ASRs and to build more accurate strategies when needed.

Our study represented the time tendency of ASRs by calculating the value of EAPCs and its 95% confidence intervals. EAPC has been proven to be an effective method for quantifying ASR tendencies within the realm of public health (13, 14). EAPC calculation depended on the published research reports.


(2) y=α+bx+e

In this Equation 2, the natural logarithm of age-standardized rates [ln (ASR)] was represented by y, and the calendar year was represented by x (15). EAPC was computed using the subsequent Equation 3:


(3) $$\mathrm{EAPC}=100\times \left(\exp\left(b\right)-1\right)$$

If both the EAPCs and the 95% CI lower bounds were above zero, the ASR exhibited an upward inclination. If both the EAPC and the 95%CI upper bounds were below zero, the ASR displayed a downward inclination. If both the EAPC and the bounds of the 95%CI were equal to zero, the ASR indicated a stable inclination. Moreover, we also calculated 95% uncertainty intervals (UIs) for each metric based on the 25th and 75th values of the 1,000 posterior distribution draws (9).

Additionally, to identify the potential influencing factors for EAPCs, we conducted Pearson’s correlation analyses to assess the association between the EAPCs of ASIR, ASDR, and ASR-DALYs with SDI values at both the national and regional levels as well as with HDI. The HDI for 2019 could represent the medical level of each country (16). When the value of *p* was <0.5, there was no statistically significant relationship between EAPCs in the ASIR, ASDR, ASR-DALYs, and HDI. The software for statistical analysis in this study was the R program (R x3.6.3), and the website is https://www.r-project.org/.

## 3 Results

### 3.1 Incidence of neonatal diseases

Across the world, the number of new cases of neonatal disorders subsided from 23,592,739 cases in 1990 to 23,532,232 cases in 2019, corresponding to a decrease of 0.26%. The ASIR of neonatal disorders presented a slightly opposite trend, increasing from 358.80 per 100,000 population to 363.30 per 100,000 population in the same study period, but the ASIR of neonatal disorders remained largely stable between 1990 and 2019. As for the gender distribution, boys were more likely to develop neonatal disorders than girls (Table 1).

**Table 1:** The incident cases and ASIR in 1990 and 2019 and its temporal trends.

Characteristics	1990		2019		1990–2019
	Incident cases No. (95% UI)	ASR per 100,000 No. (95% UI)	Incident cases No. (95% UI)	ASR per 100,000 NO. (95% UI)	EAPC No. (95% CI)
Global	23592739.05 (21925383.38–25570920.79)	358.84 (333.44–389.01)	23532231.92 (21672528.27–25701964.35)	363.34 (334.63–396.84)	0 (−0.06–0.06)
Sex					
Male	12835681.34 (11907788.96–13899857.15)	378 (350.60–409.45)	12654143.48 (11671263.37–13791272.49)	377.8 (348.46–411.77)	−0.05 (−0.12–0.02)
Female	10757057.71 (10016635.50–11668106.65)	338.38 (315.06–366.99)	10878088.44 (9970690.30–11891471.25)	347.85 (318.84–380.27)	0.05 (0.00–0.10)
Sociodemographic index					
Low	854581.69 (607921.87–1173108.68)	461.99 (435.16–496.33)	1510719.92 (1066565.83–2075843.31)	437.48 (409.15–471.42)	−0.17 (−0.22 to −0.12)
Low-middle	1364210.35 (972904.18–1857988.95)	421.35 (396.46–451.9)	1591860.72 (1132016.65–2146643.67)	390.84 (361.36–424.63)	−0.31 (−0.37 to −0.25)
Middle	1932248.07 (1355953.66–2606771.39)	324.01 (293.66–358.51)	1875710.25 (1290221.35–2558776.39)	328.45 (293.70–369.17)	0 (−0.03–0.04)
High-Middle	876242.28 (604272.88–1204923.23)	285.67 (256.63–319.62)	804064.39 (555269.00–1087955.60)	297.89 (264.78–335.49)	0.15 (0.13–0.18)
High	1177623.43 (1138401.45–1224626.36)	207.52 (200.61–215.79)	1089953.24 (1054989.21–1127074.87)	219.55 (212.54–226.99)	0.21 (0.11–0.31)
Region					
Andean Latin America	247280.61 (225027.99–269027.33)	428.27 (389.68–465.95)	238243.06 (215056.21–259154.62)	378.77 (341.91–412.02)	−0.48 (−0.58 to −0.38)
Australasia	24997.93 (23816.20–26200.21)	164.21 (156.45–172.12)	30459.39 (28790.54–32063.09)	172.42 (162.98–181.51)	0.18 (0.09–0.26)
Caribbean	187373.70 (176477.67–198703.79)	432.14 (407.01–458.26)	180206.43 (169847.97–190650.52)	461.44 (434.91–488.20)	0.17 (0.12–0.22)
Central Asia	252763.90 (233588.83–275930.48)	270.09 (249.59–294.86)	223779.72 (207893.87–240250.06)	247.21 (229.69–265.34)	−0.36 (−0.41 to −0.31)
Central Europe	186103.58 (171803.06–201869.02)	231.46 (213.71–251.03)	115806.39 (108220.46–124704.84)	224.33 (209.7–241.47)	−0.28 (−0.42 to −0.13)
Central Latin America	794628.62 (720967.9–883376.8)	334.65 (303.62–372.04)	785713.53 (699016.63–879676.08)	372.29 (331.29–416.66)	0.42 (0.33–0.5)
Central Sub-Saharan Africa	448060.87 (418733.12–480545.96)	351.27 (328.27–376.72)	726892.41 (672375.16–791568.01)	341.45 (315.84–371.84)	0.02 (−0.04–0.08)
East Asia	3096270.33 (2723273.92–3548479.26)	257.35 (226.36–294.91)	2136197.71 (1771248.44–2569015.03)	287.93 (238.83–345.89)	0.38 (0.29–0.48)
Eastern Europe	475394.22 (409425.09–552245.98)	338.13 (291.32–392.44)	336350.96 (291026.9–391204.58)	310.06 (268.39–360.67)	−0.34 (−0.38 to −0.3)
Eastern Sub-Saharan Africa	1966899.6 (1820292.78–2159174.34)	462.34 (427.92–507.49)	2846008.76 (2601338.85–3150784.88)	422.06 (385.74–467.23)	−0.30 (−0.39 to −0.22)
High-income Asia Pacific	137080.01 (131824.27–142637.2)	144.96 (139.41–150.83)	104934.32 (101365.39–108619.42)	158.44 (153.06–163.99)	0.37 (0.29–0.44)
High-income North America	547331.60 (530275.46–569419.58)	248.81 (241.07–258.86)	536707.33 (520574.49–555107.98)	266.04 (258.06–275.15)	0.22 (0.06–0.37)
North Africa and the Middle East	1907704.13 (1827432.66–1995680.67)	340.32 (325.96–356)	2102529.69 (2016483.53–2198106.45)	361.32 (346.55–377.75)	0.16 (0.13–0.18)
Oceania	28763.69 (26980.95–30749.71)	270.24 (253.48–288.88)	51008.52 (47734.38–54574.89)	259.08 (242.45–277.19)	−0.19 (−0.25 to −0.14)
South Asia	7559787.77 (7144893.08–8079884.19)	440.25 (416.11–470.46)	6629244.66 (6153256.44–7174365.68)	413.47 (383.73–447.43)	−0.27 (−0.32 to −0.22)
Southeast Asia	2382438.46 (2089402.34–2686417.91)	394.85 (346.21–445.22)	1757890.2 (1535711.25–2004844.18)	335.96 (293.54–383.19)	−0.57 (−0.60 to −0.54)
Southern Latin America	85375.32 (78347.86–92864.9)	170.13 (156.13–185.06)	78603.6 (74432.36–83455.78)	169.46 (160.45–179.91)	0 (−0.04–0.03)
Southern Sub-Saharan Africa	296390.79 (276857.02–321092.43)	402.44 (375.87–435.97)	318918.77 (296475.4–348024.6)	400.68 (372.46–437.24)	0.02 (−0.01–0.05)
Tropical Latin America	618705.42 (569401.87–679668.02)	361.63 (332.79–397.21)	579792.42 (523428.14–652126.9)	375.11 (338.70–421.77)	0.18 (0.14–0.23)
Western Europe Western	394838.87 (385359.36–404973.18)	177.20 (172.96–181.74)	365373.74 (355654.13–375560.09)	176.52 (171.82–181.43)	−0.02 (−0.04–0)
Sub-Saharan Africa	1954549.65 (1818958.88–2127940.61)	457.22 (425.44–497.90)	3387570.30 (3156763.02–3678125.4)	432.39 (402.89–469.46)	−0.23 (−0.25 to −0.21)

**Figure 1:**
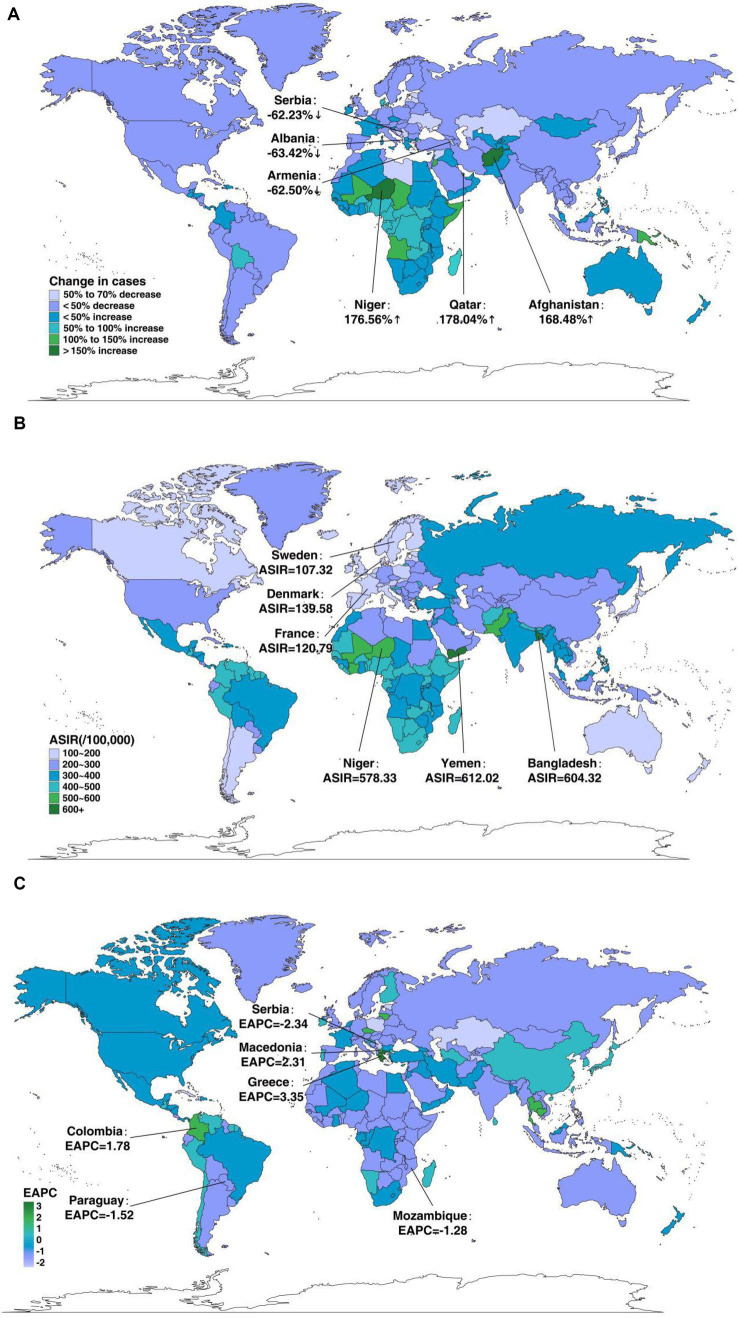
The global disease burden of neonatal disorders in 204 countries and territories. **(A)** The relative change in incident cases of neonatal disorders between 1990 and 2019. **(B)** The ASIR of neonatal disorders in 2019. **(C)** The EAPC in ASIR of neonatal disorders from 1990 to 2019.

For 204 countries, the most remarkable surge in the incidence of neonatal disorders was shown in Qatar (178.04%), with Niger (176.56%) and Afghanistan (168.48%) following closely behind, while a gross decline occurred in Albania (−63.42%), Armenia (−62.50%), and Serbia (−62.23%) (Figure 1A). The ASIR of neonatal disorders varied significantly globally in 2019, with the highest rates in Yemen (612.02/100,000, 95%UI = 583.99–642.36/100,000), followed by Bangladesh (604.32/100,000, 95%UI = 538.84–669.05/100,000) and Niger (578.33/100,000, 95%UI = 541.32–620.62/100,000), and the lowest rates in Sweden (107.32/100,000, 95%UI = 100.40–104.87/100,000), followed by France (120.79/100,000, 95%UI = 107.52–134.04/100,000) and Denmark (139.58/100,000, 95%UI = 130.5607–148.95/100,000) (Figure 1B). From 1990 to 2019, the highest rate of ASIR occurred in Greece (EAPC = 3.35, 95%CI = 3.13–3.56), followed by Macedonia (EAPC = 2.31, 95%CI = 2.12–2.51) and Colombia (EAPC = 1.78, 95%CI = 1.56–1.99), while the greatest decline in ASIR was detected in Serbia (EAPC = −2.34, 95%CI = −2.58 to 2.10), followed by Paraguay (EAPC = −1.52, 95%CI = −1.62 to 1.43) and Mozambique (EAPC = −1.28, 95%CI = −1.44 to 1.12) (Figure 1C).

Regionally, there have been great differences in ASIR over the past 30 years. Among the 21 GBD regions, the Caribbean displayed the strongest growth trend in 2019 (ASIR: 461.44/100,000, 95%UI = 434.91–488.20/100,000). The ASIR increased in 52.38% of areas (11 GBD regions) and declined in 47.62% of areas (10 GBD regions), remaining stable in one area during our study period. The largest decline and increase were observed in Southeast Asia (EAPC = −0.57, 95%CI = −0.60 to 0.54) and Central Latin America (EAPC = 0.42, 95%CI = 0.33–0.50), respectively. Among the five SDI regions, the ASIR declined in the low- and low-middle-SDI regions, while it increased in the high-middle- and high-SDI regions. The largest decline was observed in the low-middle-SDI regions (EAPC = −0.31, 95%CI = −0.37 to 0.25), while the greatest increase was observed in the high-SDI regions (EAPC = 0.21, 95%CI = 0.11–0.31). The increase in ASIR in middle-SDI regions was not obvious, with an EAPC of 0 (95%CI = −0.03 to 0.04) (Table 1).

**Figure 2:**
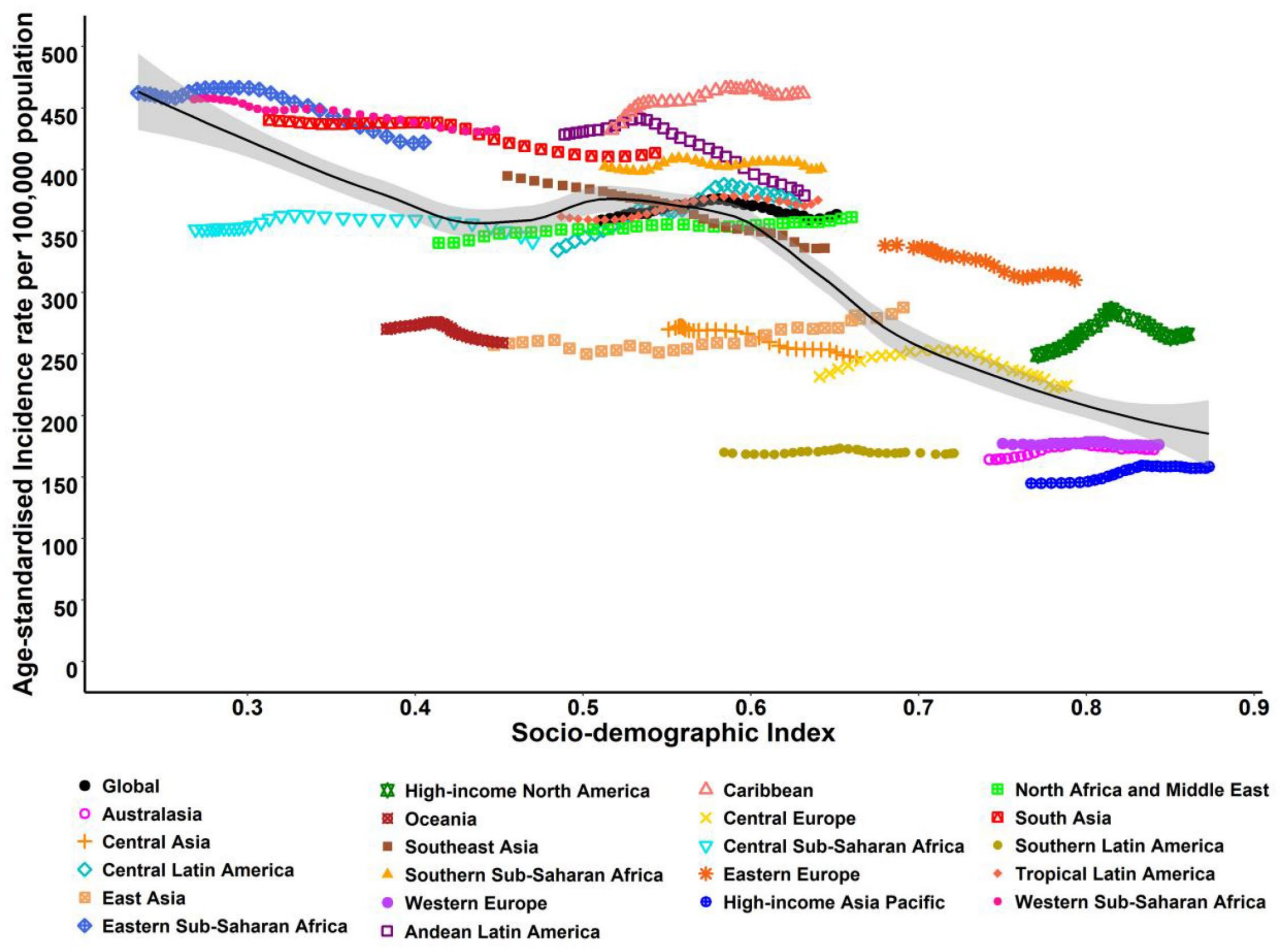
ASIR for neonatal disorders at the regional levels based on SDI in 2019 (21 GBD regions). The black line represents the expected value based on SDI and incidence rates in all locations.

In addition, in Figure 2, the correlation between ASIR and SDI at the regional and national levels in 2019 is displayed. As a whole, the ASIR and SDI were negatively related at the regional level, but when SDI was between 0.4 and 0.8, it inverted (Figure 2). Similarly, at the national level, the ASIR was also negatively correlated with SDI, with only a few exceptions (Supplementary Figure S1A).

### 3.2 Mortality of neonatal disorders

Globally, neonatal disorders caused 1,882,438 deaths (95%UI = 184, 723–201,641) in 2019, which represented a 37.38% decrease from the 3,005,945 deaths (95%UI = 2,807, 527–3,229,594) in 1990. The ASDR showed the same trend, decreasing from 45.80 per 100,000 population (95%UI = 42.80–49.20/100,000) to 29.10 per 100,000 population (95%UI = 24.80–34.50/100,000) over the course of 30 years. The gender distribution of ASDR was consistent with that of ASIR, being higher among male neonates (Table 2).

**Table 2:** The death cases and ASDR in 1990 and 2019 and its temporal trends.

Characteristics	1990		2019		1990–2019
	Death cases No. (95% UI)	ASR per 100,000 No. (95% UI)	Death cases No. (95% UI)	ASR per 100,000 No. (95% UI)	EAPC No. (95% CI)
Global	3005945.15 (2807527.40–3229594.07)	45.80 (42.78–49.22)	1882438.44 (1605827.45–2237478.10)	29.05 (24.78–34.53)	−1.51 (−1.66 to −1.36)
Sex					
Male	1681617.05 (1552952.34–1819221.73)	49.62 (45.83–53.67)	1075065.54 (906213.46–1287257.68)	32.09 (27.05–38.42)	−1.43 (−1.59 to −1.27)
Female	1324328.10 (1225946.36–1426988.64)	41.72 (38.63–44.96)	807372.90 (694761.61–944491.48)	25.80 (22.21–30.19)	−1.61 (−1.74 to −1.47)
Sociodemographic index					
Low	764713.67 (702001.03–833221.79)	68.02 (62.46–74.16)	843265.90 (697735.40–1027871.67)	46.79 (38.71–57.02)	−1.11 (−1.19 to −1.04)
Low-middle	1199828.17 (1108511.18–1299348.04)	66.36 (61.29–71.83)	674012.65 (572363.54–788390.87)	39.69 (33.7–46.42)	−1.58 (−1.73 to −1.43)
Middle	746627.87 (694094.07–800546.44)	36.16 (33.61–38.77)	281950.04 (239047.19–332818.58)	16.39 (13.89–19.34)	−2.65 (−2.88 to −2.42)
High-middle	241838.49 (221662.97–262602.2)	24.29 (22.27–26.37)	61326.19 (52383.68–71295.84)	8.07 (6.90–9.39)	−3.92 (−4.11 to −3.72)
High	51575.34 (48598.94–54848.14)	9.06 (8.54–9.64)	20809.74 (18545.73–23254.55)	4.18 (3.72–4.67)	−2.52 (−2.6 to −2.44)
Region					
Andean Latin America	22654.53 (20461.45–25112.19)	39.31 (35.50–43.57)	9907.15 (7407.72–12851.92)	15.72 (11.76–20.39)	−3.05 (−3.21 to −2.89)
Australasia	1071.40 (1001.38–1166.52)	7.02 (6.56–7.65)	587.41 (486–703.68)	3.32 (2.74–3.97)	−2.15 (−2.33 to −1.98)
Caribbean	14058.47 (12483.8–15725.65)	32.45 (28.82–36.3)	10828.55 (8175.13–13916.68)	27.72 (20.93–35.63)	−0.43 (−0.50 to −0.36)
Central Asia	26094.62 (23974.75–28216.97)	27.87 (25.60–30.14)	14814.14 (12326.28–17988.1)	16.33 (13.59–19.84)	−1.84 (−2.29 to −1.38)
Central Europe	13682.61 (13148.66–14466.1)	16.95 (16.29–17.92)	2132.07 (1674.08–2654.97)	4.11 (3.23–5.12)	−5.1 (−5.28 to −4.93)
Central Latin America	75648.77 (66487.4–84654.3)	31.88 (28.02–35.67)	26140.83 (20141.43–32734.15)	12.36 (9.52–15.47)	−3.22 (−3.30 to −3.13)
Central Sub-Saharan Africa	64800.03 (53064.69–76870.77)	51.01 (41.83–60.5)	69477.18 (57971.91–83637.07)	32.65 (27.24–39.31)	−1.35 (−1.5 to −1.21)
East Asia	300008.85 (264533.57–335091.75)	24.91 (21.97–27.83)	47138.07 (40282.23–54579.31)	6.30 (5.39–7.3)	−5.06 (−5.52 to −4.59)
Eastern Europe	22782.94 (21310.63–25399.23)	16.13 (15.08–17.99)	5229.23 (4214.46–6381.89)	4.79 (3.86–5.85)	−4.59 (−4.85 to −4.33)
Eastern Sub-Saharan Africa	256814.96 (233095.22–282428.2)	60.72 (55.12–66.75)	270671.44 (215652.24–340550.61)	40.19 (32.01–50.55)	−1.23 (−1.34 to −1.12)
High-income Asia Pacific	4955.13 (4472.05–5648.66)	5.21 (4.70–5.94)	1062.44 (934.83–1195.12)	1.59 (1.39–1.79)	−3.83 (−3.99 to −3.66)
High-income North America	20581.32 (19620.30–21702.54)	9.34 (8.91–9.85)	11912.94 (10847.8–13102.72)	5.89 (5.36–6.48)	−1.26 (−1.38 to −1.13)
North Africa and the Middle East	294511.48 (259752.35–334696.68)	52.73 (46.50–59.91)	111939.64 (93403.14–134353.06)	19.19 (16.02–23.04)	−3.35 (−3.44 to −3.26)
Oceania	3096.17 (2507.25–3735.27)	29.20 (23.65–35.26)	5055.13 (3674.89–6800.02)	25.73 (18.71–34.62)	−0.31 (−0.41 to −0.22)
South Asia	1232340.07 (1111987.61–1354911.29)	72.01 (65.01–79.16)	736596.40 (625573.52–870779.09)	45.96 (39.03–54.33)	−1.29 (−1.43 to −1.15)
Southeast Asia	234645.25 (211268.79–265989.13)	38.92 (35.05–44.13)	96717.43 (78667.06–118148.03)	18.46 (15.02–22.56)	−2.50 (−2.61 to −2.38)
Southern Latin America	11788.21 (11305.27–12285.43)	23.48 (22.51–24.46)	4014.10 (3078.21–5094.01)	8.63 (6.62–10.95)	−3.38 (−3.46 to −3.3)
Southern Sub-Saharan Africa	31951.40 (27444.04–36970.04)	43.45 (37.32–50.26)	29051.20 (22635.80–37345.25)	36.49 (28.43–46.91)	−0.35 (−0.67 to −0.03)
Tropical Latin America	83811.87 (73240.04–96421.63)	49.03 (42.85–56.4)	30209.51 (23865.93–37332.57)	19.47 (15.39–24.06)	−2.99 (−3.15 to −2.82)
Western Europe	16095.53 (15399.38–17239.14)	7.20 (6.89–7.71)	6469.62 (5397.16–7640.31)	3.11 (2.60–3.67)	−2.58 (−2.72 to −2.44)
Western Sub-Saharan Africa	274551.54 (247257.55–305938.61)	64.49 (58.08–71.85)	392483.96 (328333.22–476695.76)	50.17 (41.98–60.94)	−0.81 (−0.87 to −0.75)

In the field of 204 countries, the Cook Islands (−95.88%) saw the most substantial decline in death cases, followed by Estonia (−92.35%) and Serbia (−91.25%). Meanwhile, Somalia (106.89%) experienced the largest increase, followed by Papua New Guinea (94.64%) and Niger (90.85%) (Supplementary Figure S2A). The ASDR of neonatal disorders varied considerably across the world in 2019, with the lowest ASDR observed in Japan (1.07/100,000, 95%UI = 0.86–1.23/100,000), Singapore (1.08/100,000, 95%UI = 0.72–1.57/100,000), and the Cook Islands (1.14/100,000, 95%UI = 0.68–1.68/100,000), whereas the highest ASDR was shown in Pakistan (78.62/100,000, 95%UI = 64.03–95.20/100,000), followed by Mali (69.67/100,000, 95%UI = 55.64–87.27/100,000) and the Central African Republic (56.94/100,000, 95%UI = 43.72–73.31/100,000; Supplementary Figure S2B). Meanwhile, during the whole study period, the trend of largest decline in ASDR was discovered in the Cook Islands (EAPC = −9.04, 95%CI = −9.69 to 8.38), followed by Estonia (EAPC = −8.12, 95%CI = −8.46 to 7.77) and Saudi Arabia (EAPC = −7.38, 95%CI = −7.73 to 7.03), while the trend of greatest increase in ASDR was found in Dominica (EAPC = 1.26, 95%CI = 1.01–1.50), followed by Guam (EAPC = 1.07, 95%CI = 0.91–1.24) and Brunei Darussalam (EAPC = 0.53, 95%CI = 0.28–0.78; Supplementary Figure S2C).

With regard to geographic regions, among 21 GBD regions, Western Sub-Saharan Africa showed the most severe rates in 2019 (ASDR: 50.17/100,000, 95%UI = 41.98–60.94/100,000). However, overall, the ASDR presented a declining trend in all regions throughout the study duration, with a remarkable reduction occurring in Central Europe (EAPC = −5.10, 95%CI = −5.28 to 4.93) and East Asia (EAPC = −5.06, 95%CI = −5.52 to 4.59). Among five SDI regions, the ASDR also exhibited a downward trend across all areas, with the largest decline in high-middle-SDI regions (EAPC = −3.92, 95%CI = −4.11 to 3.72) and the smallest reduction in low-SDI regions (EAPC = −1.11, 95%CI = −1.19 to 1.04) (Table 2). Moreover, in terms of the regional and national perspective, a remarkable inverse relevance was observed between ASDR and SDI in 2019, with some exceptions (Supplementary Figures S1B, S3).

### 3.3 Disability-adjusted life year of neonatal disorder

Globally, neonatal disorder DALY cases decreased by 32.26% between 1990, when there were 274,419,931 cases, and 2019, when there were 185,886,390 cases. The ASR-DALYs declined from 4198.50/100,000 (95%UI = 3918.70–4501.30/100,000) in 1990 to 2828.30/100,000 (95%UI = 2441.60–3329.60/100,000) in 2019, with a greater decrease observed in male neonates than in female neonates (Table 3).

**Table 3:** The DALYs and age-standardized DALYs rate in 1990 and 2019 and its temporal trends.

Characteristics	1990		2019		1990–2019
	DALY cases No. (95%UI)	ASR per 100,000 No. (95%UI)	DALY cases No. (95%UI)	ASR per 100,000 No. (95%UI)	EAPC No. (95%CI)
Global	274419930.50 (256271439–294303040.10)	4198.51 (3918.68–4501.26)	185886390.1 (160501578.50–218526197.20)	2828.31 (2441.63–3329.65)	−1.30 (−1.42 to −1.18)
Sex					
Male	153098472.20 (141706237.60–165173881.90)	4535.07 (4196.08–4894.09)	104982525.5 (89992179.31–124058674.9)	3098.44 (2651.33–3663.6)	−1.24 (−1.38 to −1.1)
nFemale	121321458.3 (112482926.1–130572826.5)	3838.71 (3559.31–4131.48)	80903864.61 (70493249.11–93894238.73)	2538.58 (2212.35–2954.34)	−1.38 (−1.48 to −1.27)
Sociodemographic index					
Low SDI	68523061.05 (62939947.74–74520469.16)	6127.49 (5627.28–6664.68)	77939747.87 (65053240.52–94676453.24)	4386.53 (3671.07–5309.35)	−0.97 (−1.03 to −0.92)
Low-middle SDI	108201432.9 (99922330.12–117342968.60)	6015.63 (5553.38–6518.67)	64958149.55 (55797763.54–75326412.42)	3804.02 (3264.99–4414.23)	−1.40 (−1.52 to −1.27)
Middle SDI	68568482.40 (63873868.6–73264199.06)	3331.29 (3103.85–3559.24)	30903196.27 (26745267.21–35818934.6)	1707.85 (1475.14–1987.35)	−2.25 (−2.42 to −2.07)
High-middle SDI	23185907.85 (21327285.95–25124418.10)	2305.81 (2120.71–2498.47)	8524076.57 (7479005.36–9701687.20)	953.18 (835.90–1093.67)	−3.18 (−3.31 to −3.05)
High SDI	5815092.86 (5437077.25–6242297.33)	964.48 (905.44–1028.11)	3455360.35 (3065324.36–3877433.78)	553.11 (498.19–613.06)	−1.78 (−1.85 to −1.71)
Region					
Andean Latin America	2082533.19 (1888298.70–2302269.5)	3652.64 (3312.21–4030.48)	1091231.85 (860579.14–1361890.85)	1724.18 (1358.42–2154.87)	−2.51 (−2.61 to −2.41)
Australasia	129023.16 (118908.73–141378.20)	796.79 (738.74–869.3)	94676.58 (81362.57–108214.77)	455.39 (391.38–519.37)	−1.66 (−1.78 to −1.54)
Caribbean	1330471.26 (1186556.81–1476727.96)	3100.55 (2763.56–3445.59)	1095456.13 (852014.75–1377661.74)	2754.12 (2132.63–3472.71)	−0.29 (−0.36 to −0.22)
Central Asia	2422098.3 (2232351.65–2617320.23)	2612.84 (2412.15–2824.66)	1522377.95 (1308498.93–1811432.87)	1665.11 (1430.69–1980.63)	−1.55 (−1.94 to −1.15)
Central Europe	1411332.20 (1340602.67–1494057.18)	1673.93 (1600.56–1765.74)	370369.51 (314160.49–432383.09)	549.25 (457.94–649.84)	−4.06 (−4.21 to −3.91)
Central Latin America	7031193.44 (6232668.91–7825592.31)	2999.49 (2666.9–3333.93)	2926331.88 (2360704.44–3546600.27)	1340.34 (1073.93–1634.19)	−2.75 (−2.81 to −2.69)
Central Sub-Saharan Africa	5802030.44 (4762631.29–6872536.07)	4588.24 (3776.9–5425.52)	6488410.68 (5461238.23–7787852.96)	3098.51 (2612.94–3713.83)	−1.17 (−1.27 to −1.06)
East Asia	27797055.51 (24645212.66–31014675.11)	2303.26 (2041.91–2570.23)	7271957.51 (6376403.82–8313229.17)	786.82 (693.82–892.08)	−4.07 (−4.41 to −3.73)
Eastern Europe	2426128.74 (2248680.73–2682723.39)	1623.86 (1510.42–1797.53)	836030.28 (716986.92–958746.23)	630.7 (539.68–726.82)	−3.67 (−3.88 to −3.45)
Eastern Sub-Saharan Africa	22998589.32 (20892957.50–25241064.33)	5465.05 (4967.65–5997.52)	25303562.68 (20467578.74–31448404.61)	3826.04 (3118.38–4737.88)	−1.04 (−1.12 to −0.96)
High-income Asia Pacific	654184.58 (583706.39–739267.93)	591.65 (534.84–662.82)	320692.86 (269911.02–371914.38)	284.56 (248.76–321.99)	−2.28 (−2.43 to −2.14)
High-income North America	2347210.74 (2196285.91–2512600.29)	1025.91 (964.73–1094.13)	1725713.14 (1552834.7–1910604.99)	731.78 (663.92–803.42)	−0.89 (−0.99 to −0.78)
North Africa and the Middle East	26791130.89 (23756874.57–30400022.58)	4843.89 (4301.74–5491.25)	11489912.46 (9809947.01–13589112.4)	1950.77 (1664.11–2310.03)	−3.02 (−3.08 to −2.96)
Oceania	283282.01 (231607.48–341765.23)	2701.21 (2214.51–3254.9)	468500.83 (346400.75–621679.76)	2414.72 (1792.70–3194.85)	−0.27 (−0.36 to −0.18)
South Asia	111252819.3 (100701095.60–121998145.50)	6533.18 (5914.37–7168.1)	71241073.21 (61042999.02–83585114.25)	4395.15 (3765.20–5157.61)	−1.12 (−1.24 to −1)
Southeast Asia	21473369.22 (19388063.35–24219078.31)	3578.23 (3233.31–4032.47)	10053228.28 (8349665.37–12031297.78)	1858.88 (1542.8–2234.43)	−2.20 (−2.28 to −2.12)
Southern Latin America	1124723.58 (1079404.03–1169898.04)	2239.58 (2149.22–2329.41)	462939.50 (378389.19–566820.66)	934.32 (758–1151.48)	−2.95 (−3.03 to −2.87)
Southern Sub-Saharan Africa	2932781.13 (2538712.67–3383070.94)	4011.07 (3472.4–4623.82)	2828758.67 (2280606.24–3564928.58)	3539.88 (2849.48–4464.41)	−0.19 (−0.46–0.08)
Tropical Latin America	7655220.18 (6703646.33–8784192.32)	4482.08 (3924.67–5142.5)	3292307.49 (2722392.04–3928836.65)	2013.03 (1649.35–2431.32)	−2.58 (−2.7 to −2.46)
Western Europe	1920871.56 (1784361.68–2076092.37)	778.70 (733.88–833.3)	1145158.41 (992872.65–1311287.49)	426.39 (371.55–483.73)	−1.83 (−1.96 to −1.7)
Western Sub-Saharan Africa	24553881.80 (22134602.89–27341190.03)	5792.12 (5225.66–6448.33)	35857700.22 (30100630.31–43307004.30)	4640.71 (3904.17–5599.36)	−0.71 (−0.75 to −0.66)

**Figure 3:**
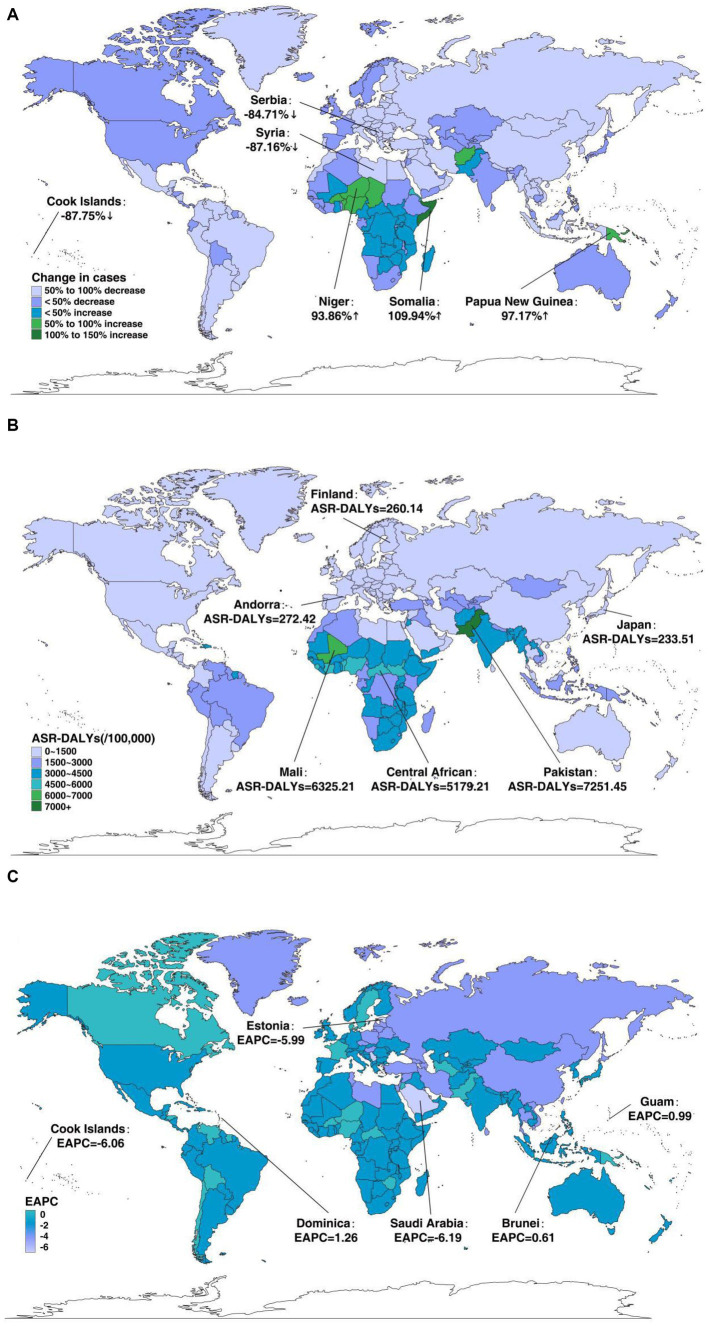
The global disease burden of neonatal disorders for both genders in 204 countries and territories. **(A)** The relative change in DALYs of neonatal disorders between 1990 and 2019. **(B)** The ASR-DALYs of neonatal disorders in 2019. **(C)** The EAPC in ASR-DALYs of neonatal disorders from 1990 to 2019.

For 204 countries, the greatest decrease in the number of DALY cases was observed in the Cook Islands (−87.75%), followed by Syria (−87.16%) and Serbia (−84.71%), while the largest increase occurred in Somalia (109.94%), followed by Papua New Guinea (97.17%) and Niger (93.86%) (Figure 3A). The ASR-DALYs considerably varied in different countries during the study period. In 2019, the ASR-DALYs were lowest in Japan (233.51/100,000, 95%UI = 198.29–267.77/100,000), followed by Finland (260.14/100,000, 95%UI = 210.77–307.63/100,000) and Andorra (272.42/100,000, 95%UI = 223.04–331.59/100,000), while they were highest in Pakistan (7251.45/100,000, 95%UI = 5979.99–8714.79/100,000), followed by Mali (6325.21/100,000, 95%UI = 5072.35–7894.88/100,000) and Central African Republic (5179.21/100,000, 95%UI = 4002.43–6622.66/100,000; Figure 3B). As for annual tendency, Saudi Arabia (EAPC = −6.19, 95%CI = −6.42 to 5.96) demonstrated the most significant reduction in ASR-DALYs, followed by the Cook Islands (EAPC = −6.06, 95%CI = −6.37 to 5.76) and Estonia (EAPC = −5.99, 95%CI = −6.28 to 5.69), while Dominica (EAPC = 1.26, 95%CI = 1.03–1.49) experienced the biggest growth, followed by Guam (EAPC = 0.99, 95%CI = 0.85–1.12) and Brunei Darussalam (EAPC = 0.61, 95%CI = 0.40–0.82; Figure 3C).

**Figure 4:**
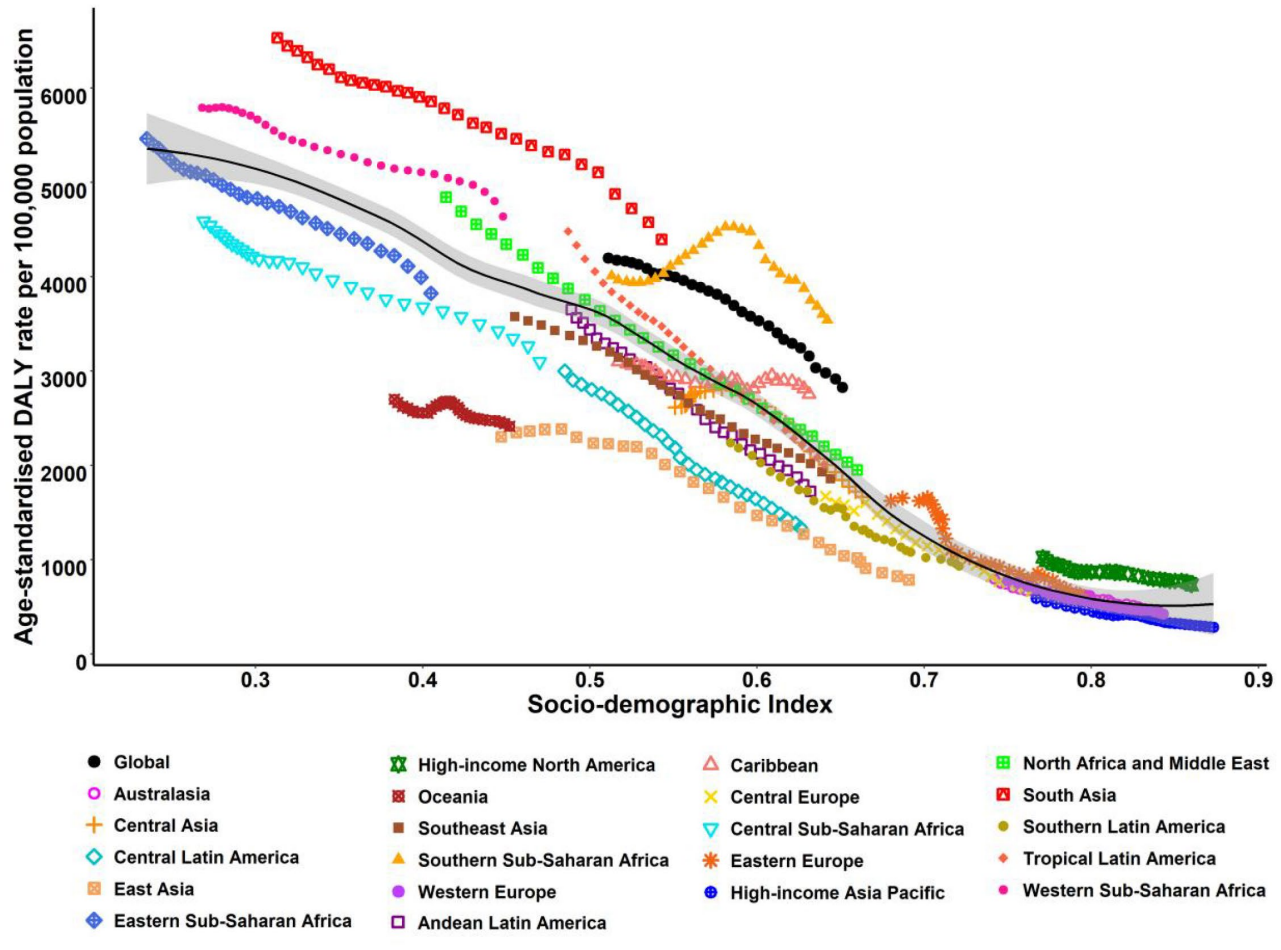
ASR-DALYs for neonatal disorders at the regional levels based on SDI in 2019 (21 GBD regions). The black line represents the expected value based on SDI and DALYs rates in all locations.

Regionally, ASR-DALYs of neonatal disorders also varied widely among regions between 1990 and 2019. Among 21 GBD areas, in 2019, the ASR-DALYs were highest in Western Sub-Saharan Africa (4640.71/100,000, 95%UI = 3904.17–5599.36/100,000) and lowest in high-income Asia Pacific (284.56/100,000, 95%UI = 248.76–321.99/100,000). Meanwhile, the ASR-DALYs declined across all regions, with the largest decrease in East Asia (EAPC = −4.07, 95%CI = −4.41 to 3.73) and Central Europe (EAPC = −4.06, 95%CI = −4.21 to 3.91). Among the five SDI regions, the ASR-DALYs also presented a decreasing tendency across all regions, with the largest decrease in the high-middle-SDI regions (EAPC = −3.18, 95%CI = −3.31 to 3.05) and the low-SDI regions having the smallest decrease (EAPC = −0.97, 95%CI = −1.03 to 0.92; Table 3). Additionally, in 2019, there was an inverse correlation between ASR-DALYs and SDI in regional and national terms, with some exceptions (Figure 4; Supplementary Figure S1C).

### 3.4 The association between HDI and EAPCs in ASIR, ASDR, and ASR-DALY of neonatal disorders

**Figure 5:**
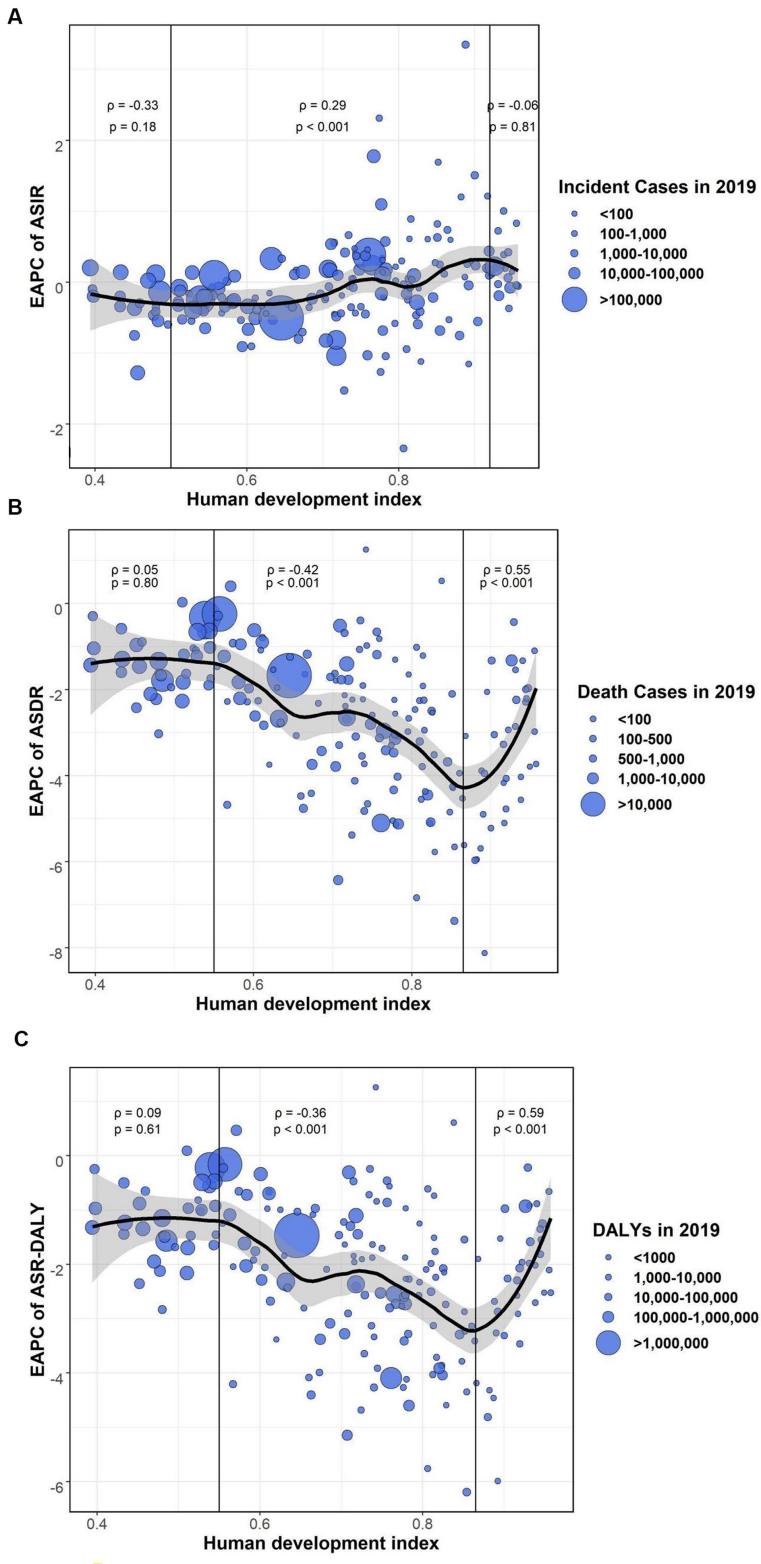
The association between HDI (2019) and EAPCs in ASIR, ASDR, and ASR-DALYs of neonatal disorders. **(A)** HDI (2019) was positively associated with EAPC in ASIR (*ρ* = 0.29, *p* < 0.001). **(B)** HDI (2019) was negatively associated with EAPC in ASDR (*ρ* = −0.42, *p* < 0.001). **(C)** HDI (2019) was negatively associated with EAPC in ASR-DALYs (*ρ* = −0.36, *p* < 0.001). The circles represent countries that were available on HDI data. The size of the circle increases with the number of cases of neonatal disorders.

There was a remarkable positive relationship between the EAPCs in ASIR and HDI in 2019 (*ρ* = 0.29, *p* < 0.001), with the HDI between 0.50 and 0.92. Conversely, when the HDI was between 0.55 and 0.87, a remarkable negative relationship was observed between the HDI and EAPCs in ASDR (EAPC = −0.42, *p* < 0.001) and ASR-DALYs (2*ρ* = −0.36, *p* < 0.001). Surprisingly, there was a strong correlation between EAPCs in the ASDR, DALYs, and HDIs in countries with higher HDI (Figure 5). In addition, nations with elevated levels of HDI have observed a faster increase in ASDR and ASR-DALYs.

## 4 Discussion

To the best of our knowledge, our study was the first to analyze the global perspective, regional and national discrepancies on the incidence, death, DALYs, and ASRs and their long-term tendency of neonatal disorders and the connection between socioeconomic status and human development index from regional and national perspectives, using the 2019 GBD data. Research results could provide policymakers across various nations or regions with the development trend of neonatal disease burden so that they could pinpoint zones of accomplishment, stagnation, and emerging risks related to neonatal disorders and learn from the progressive experience of countries or regions with well-managed disease burden.

On a global scale, we found that the overall trend of ASIR for neonatal disorders remained basically unchanged from 1990 to 2019, perhaps due to a considerable disparity in population, an unbalanced distribution of medical resources, an imperfect healthcare system, and indigenous social economy levels (17–19). This current study found that the ASDR and ASR-DALYs of neonatal disorders decreased to various degrees worldwide. This is largely in line with the previous study and is probably impacted by the improvements in maternal and newborn health services (20). Meanwhile, the burden of neonatal disorders in the male population was higher than that in the female population. Nonetheless, we should not ignore the burden of neonatal disorders in women and their related hazard factors in various regions.

Our study confirmed that the burdens of neonatal disorders had a certain significant association with social and economic development status. The burdens of neonatal disorders were not balanced in five SDI subareas, which might be due to unequal access to health protection and medical resources (21). The greatest decline in ASDR and ASR-DALYs was noticed in the high-middle-SDI regions, which was inseparable from reasonable input in the enhancement of the medicare health systems, continued medical investment, and comprehensive neonatal care (2). By comparison, the ASDR and ASR-DALYs showed the smallest decrease in low-SDI regions at the same research time. Neonatal disorders not only cause significant economic costs to low-SDI areas but also exacerbate the inequalities of social welfare (22), which becomes a vicious cycle. Notably, our investigation discovered that the highest decrease in the ASIR of neonatal disorders in low-middle-SDI regions was likely due to the significant role played by private healthcare departments in offering maternal and newborn healthcare services in countries with low to moderate incomes (23). In addition, the ASIR of neonatal disorders displayed an upward inclination in high-SDI regions. There were several potential reasons to partially but not fully explain this ascending tendency of ASIR in high-SDI regions, including the unceasing growth of the population, the increasing proportion of women of advanced reproductive age, and the continuous use of assisted reproduction techniques.

The burden of neonatal disorders varied greatly in different regions. Central Latin America showed an upward trend in ASIR from 1990 to 2019, with the most likely cause being not achieving pervasive health coverage levels in relation to reproduction and maternal and children’s health interventions especially in contemporary birth control methods, prenatal care, and trained birth attendants, and this inequality was more common among indigenous women (24). Meanwhile, the nation with the most significant decline in ASIR was Southeast Asia. One previous study in Southeast Asia reported that implementing the continuum of care (CoC) was deemed an effective method to enhance the conditions of maternal and neonatal health outcomes (25), and other studies also indicated that CoC has become a key strategy to markedly diminish the hazards of maternal, perinatal, and infant death, particularly those caused by preventable factors (26). Furthermore, our research reported that the ASDR and ASR-DALYs of neonatal disorders declined in all geographical areas, with the most substantial decrease in East Asia, which was consistent with previous findings, where mothers and babies have often been exposed to an elevated risk of infection due to insufficient hospitals and water, sanitation, and hygiene (WASH) services and gaps in hygiene practices at childbirth (27). It is well known that the importance of hygiene practices during childbirth and the postpartum period is well recognized.

The relative changes in the incidence, death cases, and DALY cases of neonatal disorders have varied dramatically among different countries since 1990 and 2019. Some countries (e.g., Qatar and Somalia) experienced significant increases in the incidence, death rate, and DALYs. Qatar was a country with a prosperous population, of which 90% were expatriates from different nationalities. Surprisingly, Qatar’s national development strategy focused only on the Qatari population. Therefore, Qatar’s long-term residents might face greater challenges in healthcare, which might also be one of the biggest reasons for the increase in the incidence rate of neonatal disorders in Qatar (28). Somalia was also facing the same problems. It was ranked as the most challenging country for mothers and had the highest rate of deaths among children under the age of 5 years globally (29), which is consistent with the results of this study. Due to crises of conflict-related and widespread drought over the past 30 years, maternal and neonatal healthcare in Somalia was facing a serious healthcare crisis (30, 31). With such a background, local health authorities in Somalia were facing the dual burden of rebuilding the healthcare system and handling urgent public health events resulting from continuing instability, conflicts, and natural disasters. As a result, the public health sector in Somalia further deteriorated to almost non-existent (32, 33). Herein, further exploring partnerships between government and service providers in the non-governmental sector is essential to provide cost-free essential maternal and child healthcare services to countries such as Somalia, which are in unsafe and turbulent environments. In addition, other countries (e.g., Albania and the Cook Islands) showed a decreasing tendency in the incidence, death rate, and DALYs. Early Essential Newborn Care (EENC) (34), international healthcare projects (35), and studies on reproductive health (36) might contribute to the downswing of the burden of neonatal disorders in the abovementioned island nations.

This research revealed that the ASIRs, ASDRs, and ASR-DALYs of neonatal disorders demonstrated a huge difference between nations in 2019. The ASIR, ASDR, and ASR-DALYs of neonatal disorders were lowest in Sweden and Japan, the possible reason being that the developed countries allocated a large number of personnel and financial assets to protect the health of mothers and children. For instance, Sweden had the best social welfare and healthcare systems in the world and led the world in terms of both diagnosis technology and treatment technology for neonatal disorders. Japan had a similar situation. It was also a country with higher welfare and perfect health and welfare among mothers and children, and it had a sound social security system, including social insurance, public medical care, and public assistance. Our study also found that the ASIR of neonatal disorders was highest in Yemen as a result of a large-scale epidemic that occurred in the protracted armed conflict environment of women and children, where the primary healthcare needs were not met (37, 38). The ASDR and ASR-DALYs were highest in Pakistan. Previous similar findings have already reported that the following key factors might explain the higher burden of neonatal disorders in Pakistan. First, a lack of formal education in Pakistan was the main reason for pregnant women experiencing difficulty in accessing key maternal health services and the main cause of worse maternal and child health outcomes (39–41). Second, malnutrition of reproductive-aged women, especially for women who are deficient in prenatal vitamin/calcium/iron supplementation or have insufficient weight gain during gestation, was strongly linked with adverse outcomes for newborns. In addition, the quality of delivery care, the delay in referrals from one healthcare facility to another, the inter-delivery interval, and newborn care seemed to be substantially worse in Pakistan (39, 42). To summarize, without addressing the aforementioned factors emphasized in this article, it was impossible for every country to achieve the ambitious mortality targets.

Our research also identified trends in the burden of illnesses affecting neonates. During the period spanning 1990 to 2019, the frequency of neonatal disorders increased most significantly in Greece and Macedonia. In these regions, the economic crisis that occurred in 2008, as well as the subsequent cost-cutting measures taken in healthcare expenditures, brought about remarkable adverse impacts on perinatal outcomes (43, 44), showing that government investment in financial and material capabilities was essential for the prevention of neonatal disorders. The frequency of perinatal and neonatal disorders was very high (45), mainly because adolescent mothers at puberty were relatively common in this country, and maternal care was also affected by racism and discrimination (46). Similarly, over the last 30 years, the countries with the highest increase in EAPCs in the ASDR and ASR-DALYs of neonatal disorders were Dominica, Guam, and Brunei, indicating that the prevention and treatment capacity of developed countries for neonatal disorders is stronger than that of underdeveloped countries. By comparison, the countries with the highest decrease in EAPCs in the ASDR and ASR-DALYs of neonatal disorders were the Cook Islands, Estonia, and Saudi Arabia. For example, Saudi Arabia has increased its investment in healthcare since 2010, and the domestic government’s general health expenditure has increased by almost 68% (47). It can be seen that the government’s approach to maternal health policies is an important link in preventing neonatal disorders. In addition, this study uncovered a negative correlation between ASIR for neonatal disorders and SDI from the nation’s point of view, with some exceptions. Similar patterns were observed for ASR-DALYs and ASDR in relation to SDI. Studies have also highlighted that countries in medically resource-poor settings still face the growing challenges of maternal and neonatal disorders (48).

Although the data acquired from the GBD study bridged the lacuna in neonatal disorders burden, this study still has some limitations. First, this study failed to conduct a statistical analysis of the types of neonatal disorders. Second, there is no analysis of the role of specific risk factors for neonatal disorders, which may help explain the geographical and temporal patterns of disease burden. Third, due to the unavailability of official data for all countries between 1990 and 2019, our study mainly focused on ASIRs, ASDRs, and ASR-DALYs’ EAPCs, and subsequent research will explore alternative data sources to refine our understanding of disease burden dynamics. Finally, this study has only obtained the impact of neonatal disorders borne by different nations or regions and cannot determine the equivalent causes.

## 5 Conclusion

To sum up, the ASIR of neonatal disorders remained steady globally during the study period, while the ASDR and ASR-DALYs displayed a more modest decline on the whole in this period, but the differences between countries and regions needed to be taken into account. Whether in high- or low-resource countries, neonatal disorders remained a noteworthy issue in children. The findings of quantitative changes in the burden trends of neonatal disorders since 1990 suggested that a dynamic effect of the adjustment of healthcare policy, the occurrence of crises that compromise safety, and the reduction of government medical expenditure on neonatal health was likely. It was necessary to implement a multi-pronged approach to formulate current prevention strategies and build more pertinent and concrete tactics in countries with relatively higher ASIR, ASDR, or ASR-DALYs to prevent and control the increasing burden of neonatal disorders.

## Data availability statement

The original contributions presented in the study are included in the article/Supplementary material, further inquiries can be directed to the corresponding author.

## Author contributions

JX: Writing – original draft, Writing – review & editing. YL: Writing – original draft, Writing – review & editing. WX: Writing – original draft, Writing – review & editing. SZ: Writing – original draft, Writing – review & editing. HN: Writing – original draft, Writing – review & editing. JF: Writing – original draft, Writing – review & editing. LS: Writing – original draft, Writing – review & editing. BZ: Writing – original draft, Writing – review & editing. XZ: Writing – original draft, Writing – review & editing. WY: Writing – original draft, Writing – review & editing.
