# Supplementary figures and images for: Measurement of the burdens of neonatal disorders in 204 countries, 1990–2019: a global burden of disease-based study
Source: Front Public Health. 2024 Jan 9;11:1282451. doi: 10.3389/fpubh.2023.1282451 (PMC10803531; doi:10.3389/fpubh.2023.1282451)

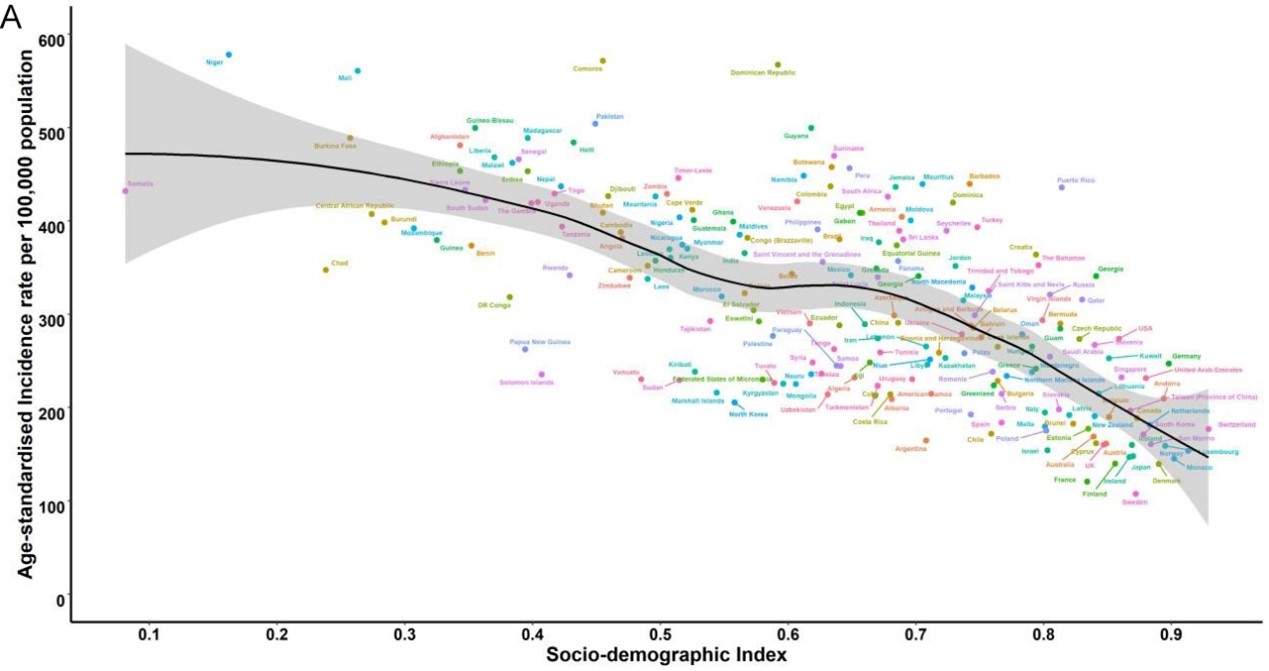

Supplement: Supplementary file 1 [file Image_1.jpg]

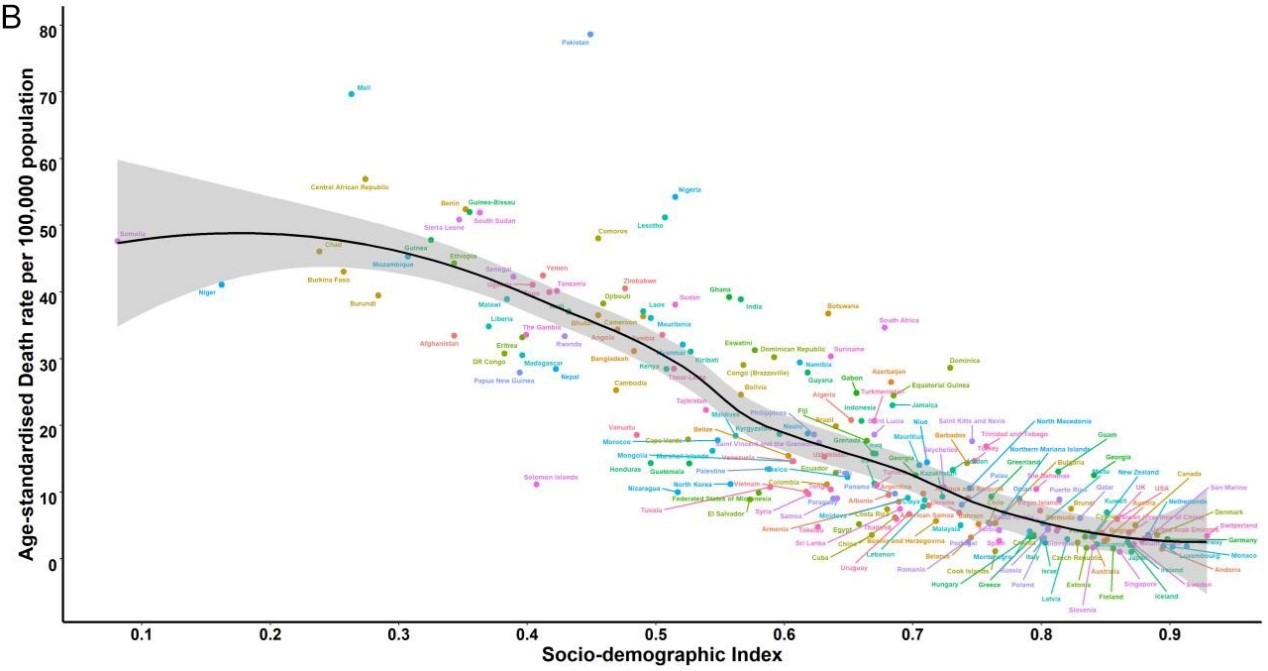

Supplement: Supplementary file 2 [file Image_2.jpg]

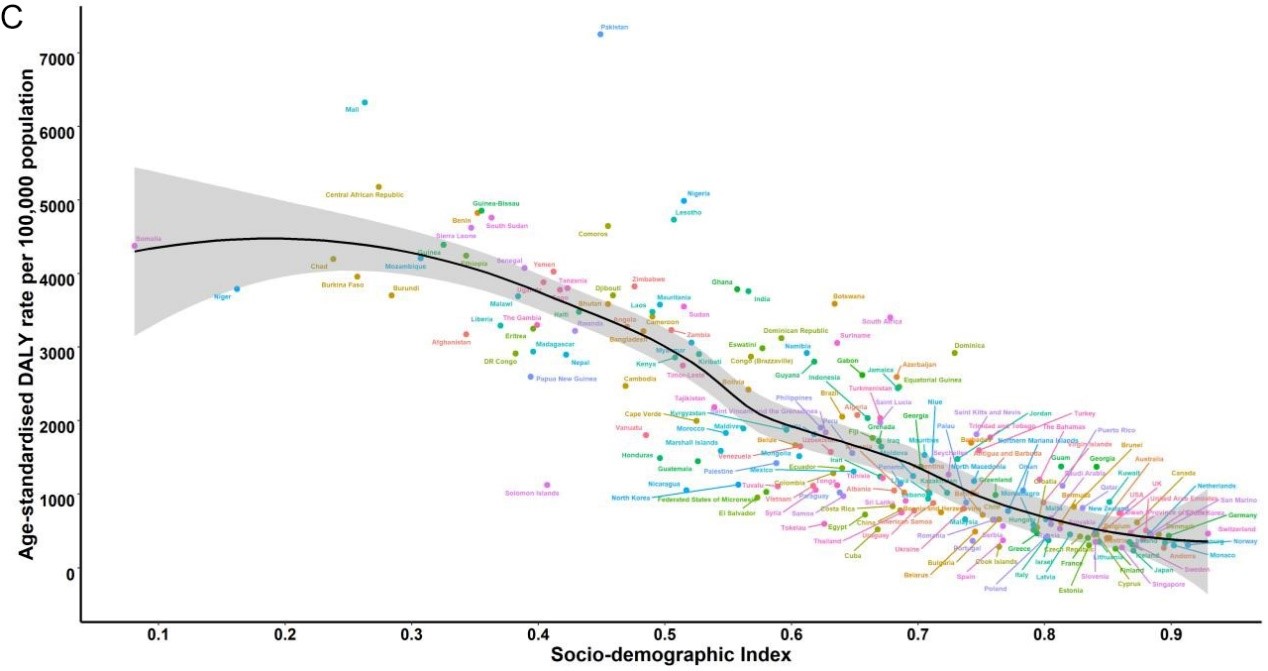

Supplement: Supplementary file 3 [file Image_3.jpg]

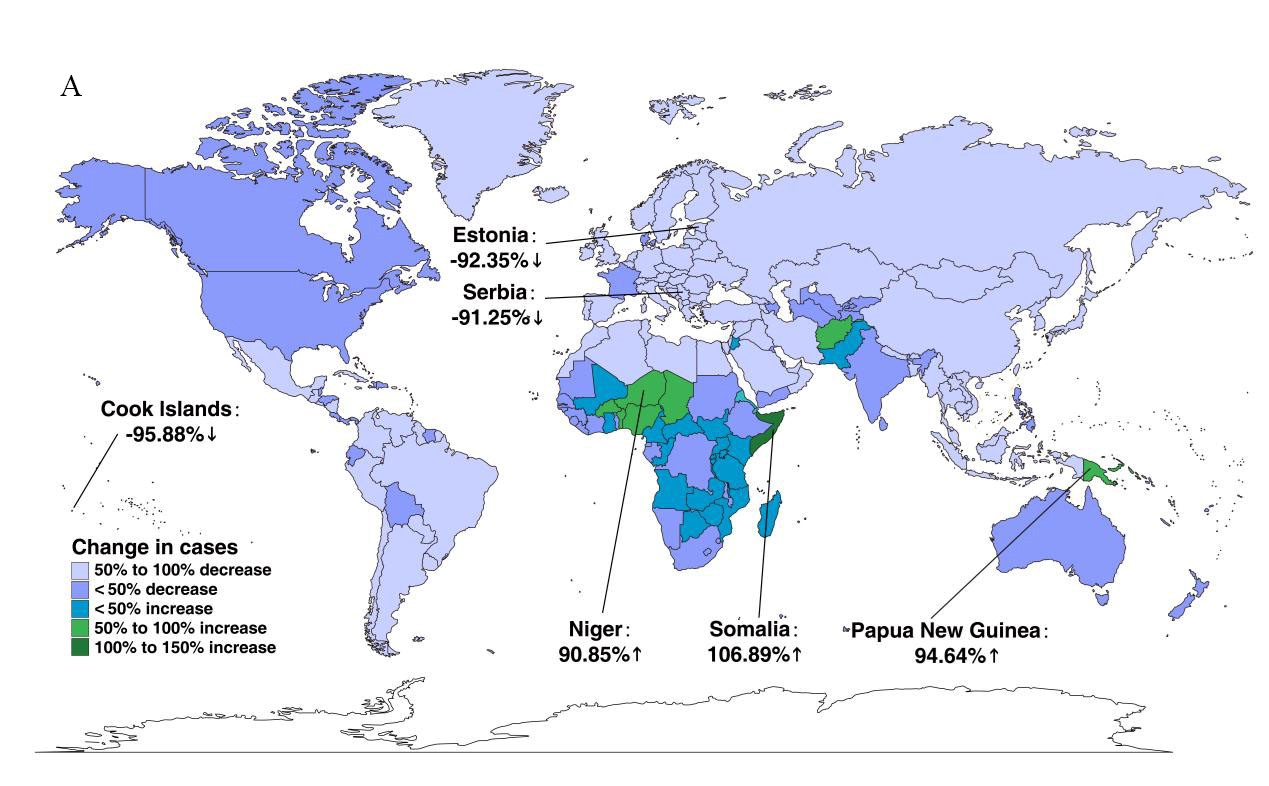

Supplement: Supplementary file 4 [file Image_4.jpg]

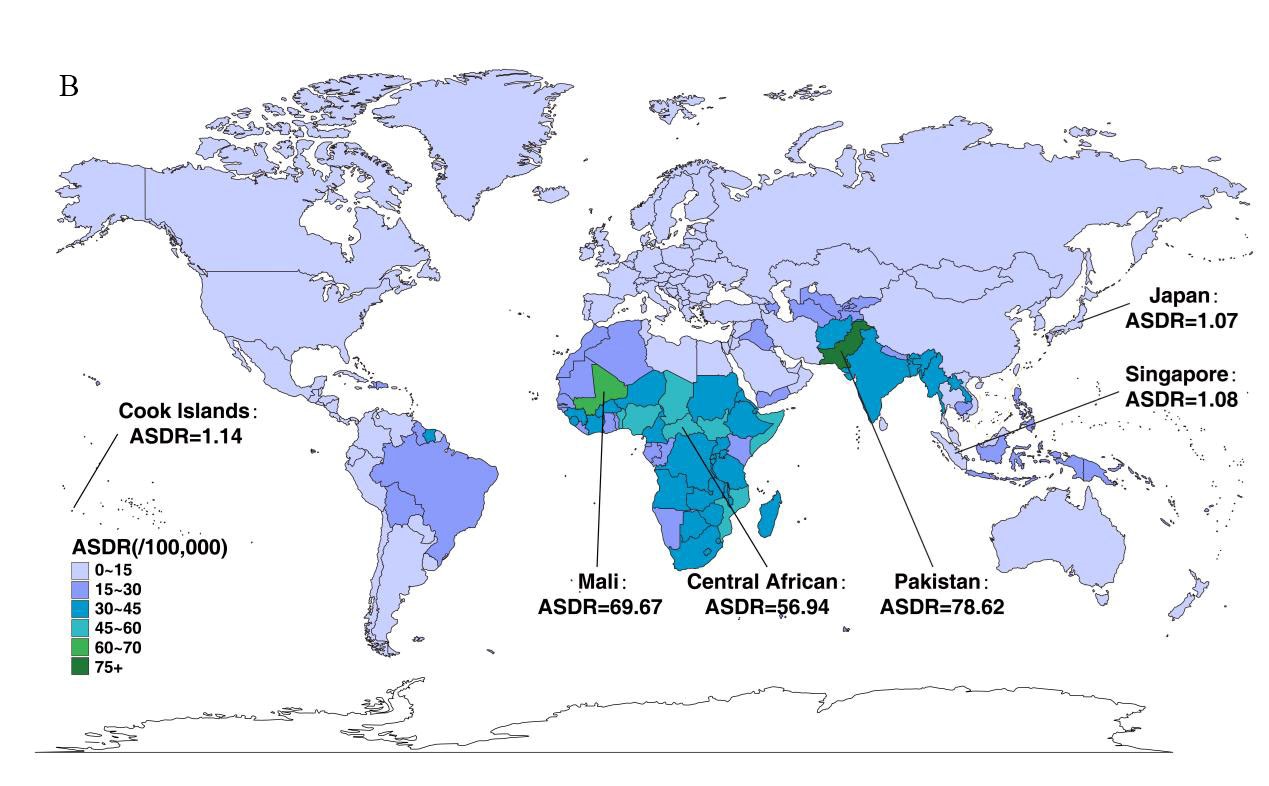

Supplement: Supplementary file 5 [file Image_5.jpg]

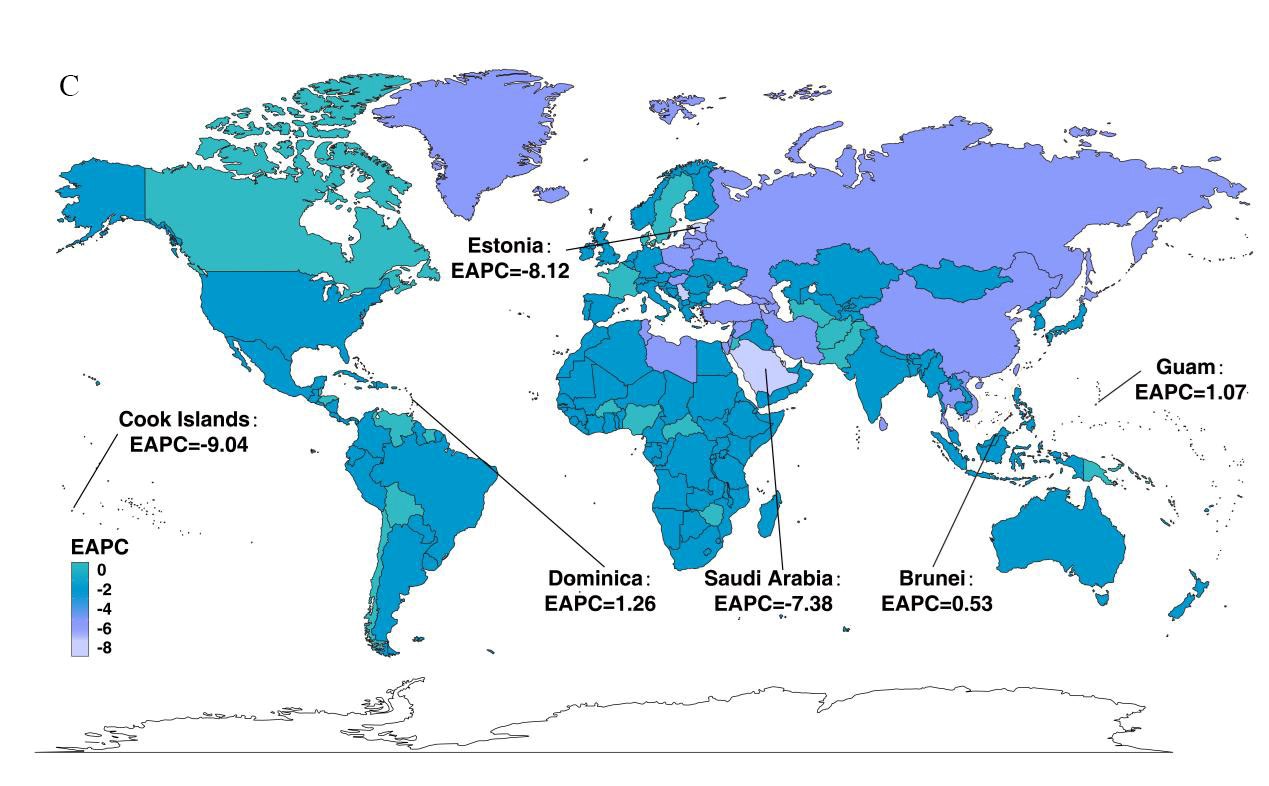

Supplement: Supplementary file 6 [file Image_6.jpg]

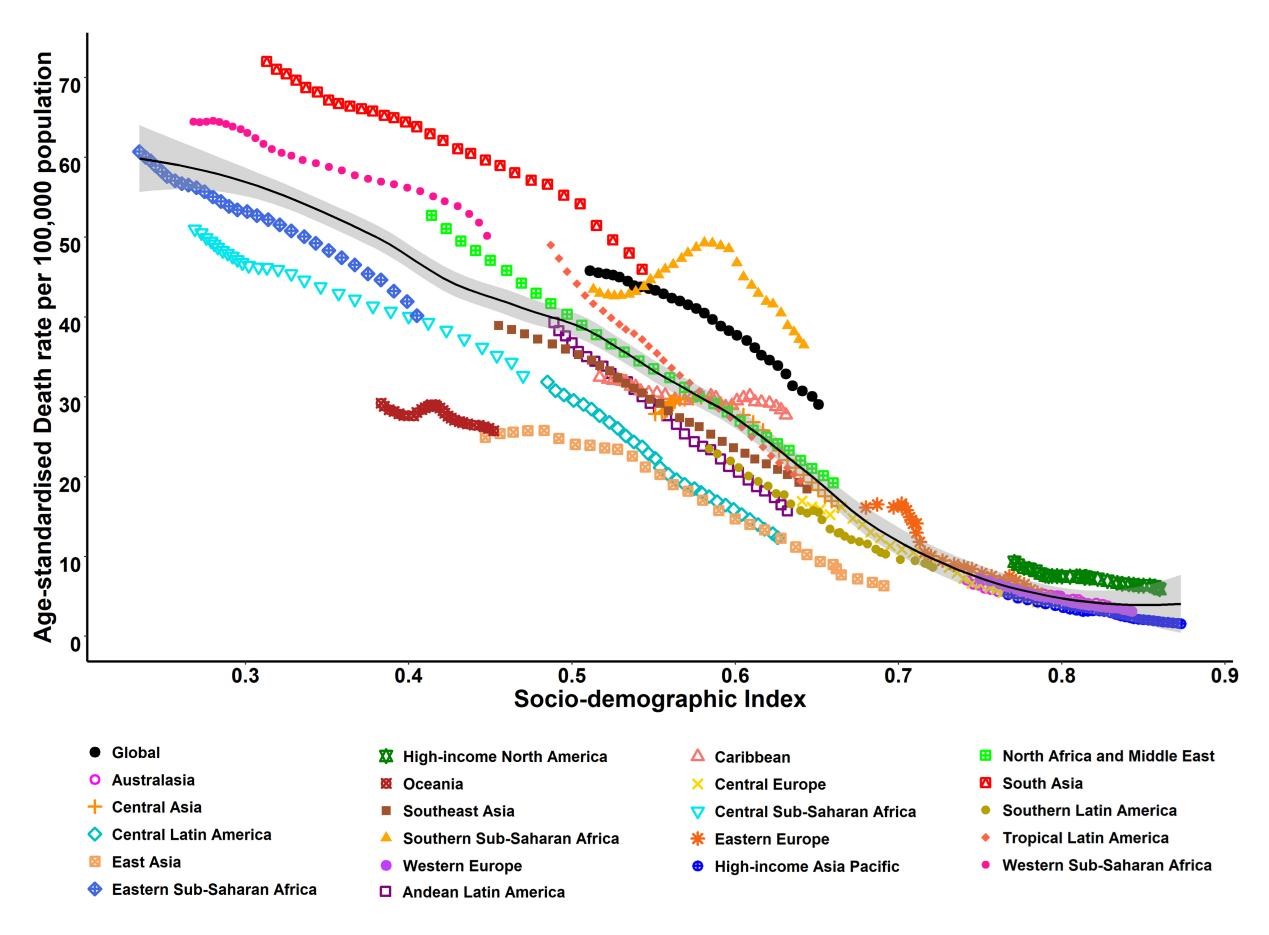

Supplement: Supplementary file 7 [file Image_7.jpg]
